# A Brain Unfixed: Unlimited Neurogenesis and Regeneration of the Adult Planarian Nervous System

**DOI:** 10.3389/fnins.2017.00289

**Published:** 2017-05-23

**Authors:** David D. R. Brown, Bret J. Pearson

**Affiliations:** ^1^Program in Developmental and Stem Cell Biology, The Hospital for Sick ChildrenToronto, ON, Canada; ^2^Department of Molecular Genetics, University of TorontoToronto, ON, Canada; ^3^Ontario Institute for Cancer ResearchToronto, ON, Canada

**Keywords:** regeneration, planarian, adult neurogenesis, brain plasticity, stem cell heterogeneity

## Abstract

Powerful genetic tools in classical laboratory models have been fundamental to our understanding of how stem cells give rise to complex neural tissues during embryonic development. In contrast, adult neurogenesis in our model systems, if present, is typically constrained to one or a few zones of the adult brain to produce a limited subset of neurons leading to the dogma that the brain is primarily fixed post-development. The freshwater planarian (flatworm) is an invertebrate model system that challenges this dogma. The planarian possesses a brain containing several thousand neurons with very high rates of cell turnover (homeostasis), which can also be fully regenerated *de novo* from injury in just 7 days. Both homeostasis and regeneration depend on the activity of a large population of adult stem cells, called neoblasts, throughout the planarian body. Thus, much effort has been put forth to understand how the flatworm can continually give rise to the diversity of cell types found in the adult brain. Here we focus on work using single-cell genomics and functional analyses to unravel the cellular hierarchies from stem cell to neuron. In addition, we will review what is known about how planarians utilize developmental signaling to maintain proper tissue patterning, homeostasis, and cell-type diversity in their brains. Together, planarians are a powerful emerging model system to study the dynamics of adult neurogenesis and regeneration.

## Introduction

The adult brain has long been thought to be a fixed structure due to its immense complexity as is illustrated succinctly in the following quote from prominent nineteenth century neuroscientist and Nobel laureate Santiago Ramón y Cajal:

“*Once the development was ended, the founts of growth and regeneration of the axons and dendrites dried up irrevocably. In the adult centers, the nerve paths are something fixed, ended, and immutable. Everything may die, nothing may be regenerated. It is for the science of the future to change, if possible, this harsh decree.”*

Although, genetic laboratory model organisms have taught us volumes about neural development, they do not strongly challenge the view of Ramón y Cajal as adult organisms. For example, the roundworm *C. elegans* has no known adult neurogenesis. Adult *Drosophila* have little to no cell division in the adult brain, although this is (amazingly) still controversial (Ito and Hotta, 1992; von Trotha et al., 2009; Fernández-Hernández et al., 2013). Finally, mouse and human have limited neurogenesis in the sub-ventricular zone (SVZ) of the cortex and in the dentate gyrus (DG) (Altman and Das, 1965, 1967; Doetsch et al., 1999), resulting in a neuronal turnover of ~1–2% per year in humans (Spalding et al., 2013). Correlative to the paucity of adult neurogenesis, these model systems also have extremely limited neural-regenerative capacity (Cebrià, 2007).

As new experimental model systems can be functionally interrogated with CRISPR/Cas9 technology, as well as molecular and genomic techniques, the dogma of Ramón y Cajal is now being challenged. In fact, high levels of adult neurogenesis and neural regeneration have been found in cnidarians, invertebrates, and vertebrates alike, suggesting that the ancestral state was that of significant neural plasticity (Holstein et al., 2003; Reddien and Sánchez Alvarado, 2004; Tanaka and Reddien, 2011; Kizil et al., 2012; Ross et al., 2017). Highly-regenerative organisms that can replace much of their nervous system offer a unique opportunity to study the cellular and molecular underpinnings of adult neurogenesis in an unrestricted context. For example, zebrafish are vertebrates that demonstrate adult neurogenesis along the entire rostral-caudal axis of the brain (Grandel et al., 2006) and have some ability to regenerate damaged regions of their central nervous system (CNS; brain and spinal cord; Kroehne et al., 2011). Although, humans have limited capacity to generate adult neurons, it has been demonstrated that transplanted neurons are capable of integrating into the brain (Falkner et al., 2016). Despite this finding, proper and full functional integration of neurons into the adult brain has been problematic and requires refinement before therapies can be effective (Brundin et al., 2010). It is currently thought that if we can understand the biology of adult neurogenesis and neural regeneration in other model systems, we will be able to drive a patient's own cells to become hyper-regeneration-competent and differentiate into neurons that can integrate into existing neural circuitry (Kim et al., 2013). Thus, inducing adult neurogenesis and cell integration in humans may hold the potential to regenerate and heal a brain after disease or injury.

**Figure 1:**
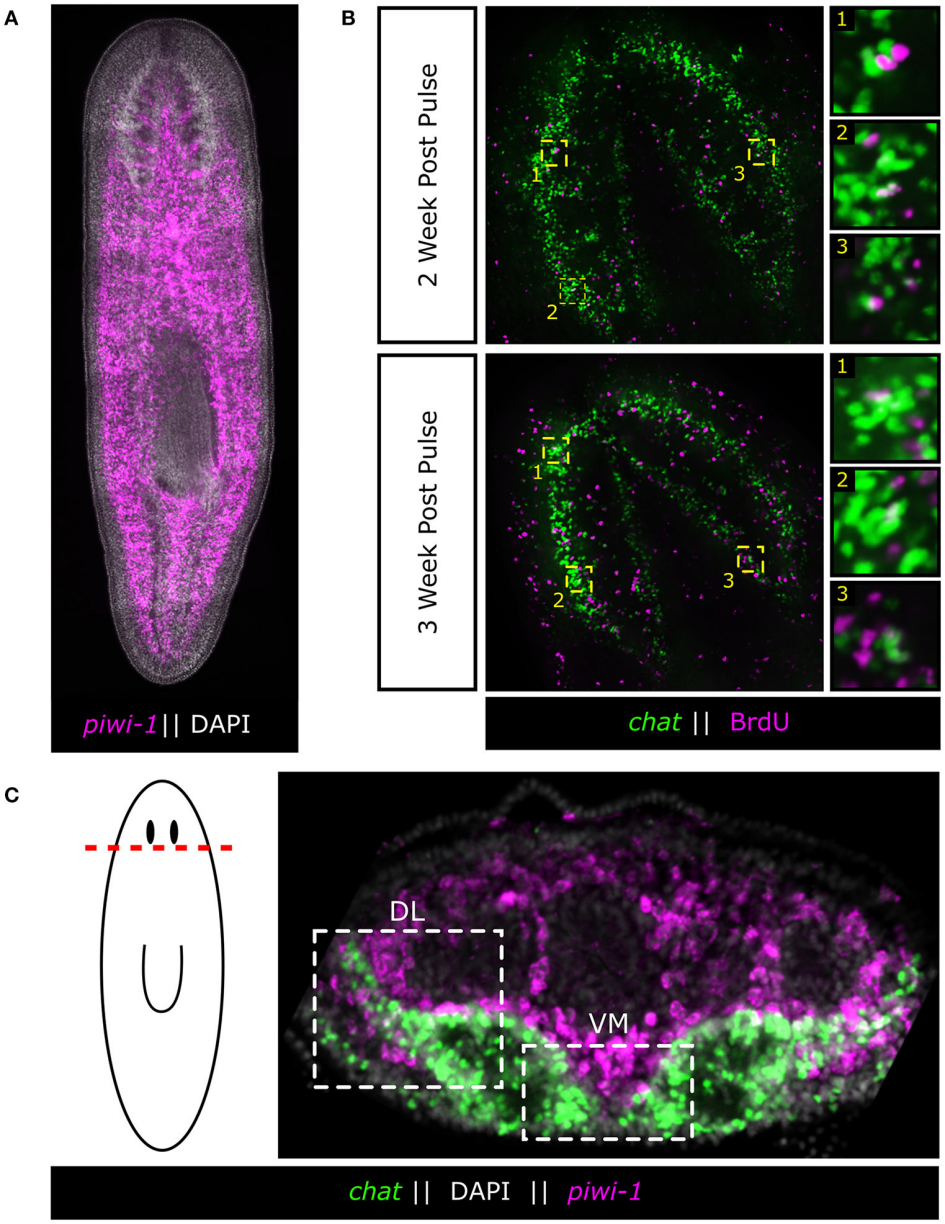
**Adult neurons are born continuously in the adult flatworm from a large population of adult stem cells. (A)** Fluorescent *in situ* hybridization (FISH) of planarian adult stem cells (neoblasts) as marked by *piwi-1*. Neoblasts span the entire length of the body except for the region anterior to the eyes. **(B)** BrdU pulse chase experiments demonstrating the continuous nature of adult neurogenesis in the planarian. Worms were fed BrdU food (25 mg/mL) 2 days in a row and chased for 2 and 3 weeks. BrdU was detected and combined with FISH of the brain marker *chat* to mark acetylcholine neurons. Panels are representative single slice confocal planes. **(C)** Adult stem cells surround the Ventral-Medial (VM) and Dorsal-Lateral (DL) zones of the brain, two putative neurogenic zones. These stem cells are the likely source of new neurons during adult neurogenesis.

Perhaps the most powerful CNS-regenerator in the laboratory is the invertebrate freshwater planarian, *Schmidtea mediterranea*, which is a flatworm of the phylum Platyhelminthes and is capable of limitless regeneration (Tan et al., 2012). The asexual laboratory strain is an extremely long-lived, constitutive adult animal with a brain possessing on the order of 1,000–10,000 cells, which can completely regenerate its whole brain and functionally reintegrate the new tissue in ~7 days without scaring (Cebrià, 2007). In addition, the planarian brain can be rescaled in size and proportion as the animal grows or shrinks, which can be a ~10-fold change in length and ~100-fold change in area (Newmark and Sánchez Alvarado, 2002; Hill and Petersen, 2015). Finally, the planarian brain undergoes constant neuronal turnover of ~25% per week (Figure 1; Zhu et al., 2015; Currie et al., 2016). Altogether, the planarian brain may represent the most dynamic adult CNS found in animals.

Planarians owe their regenerative ability and neuronal turnover (homeostasis) to a large population of somatic stem cells, called “neoblasts,” which populate the mesenchyme of the worm and account for ~20% of all cells in the animal (Baguñà et al., 1989; Sánchez Alvarado and Kang, 2005; Baguñà, 2012; Zhu and Pearson, 2016; Figure 1). As far as has been examined, every tissue in the adult animal undergoes some level of turnover without injury, and every tissue can be regenerated. However, it should be noted that the regulators of cellular lifespan and whether dying cells secrete signals, are completely unknown. Thus, the field has recently focused on the tissue-specific nature of the stem cells and whether or not heterogeneity exists (Reddien, 2013). To this end, several key questions are raised when considering a long-lived flatworm's potential for continuous adult neurogenesis: (1) How does the planarian dedicate a sub-fraction of its large stem cell population to neural homeostasis in order to generate the diversity of neuronal subtypes found in the adult flatworm? and (2) How does the planarian dynamically modulate neurogenesis to entirely regenerate or maintain scale and proportion of the adult brain?

Here we will highlight candidate-based functional and single-cell sequencing studies that have focused on characterizing the heterogeneity that exists within the planarian stem cell pool to drive the continual generation of a plethora of neuronal subtypes. Additionally, we will review what is known about how planarians utilize conserved developmental signaling pathways to define neurogenic zones that dynamically couple neoblasts, capable of continuous and unlimited adult neurogenesis, with mature and fully patterned neurons of the adult brain.

## From neoblast to neuron: stem cell heterogeneity underpins adult neurogenesis

Although, planarians possess a massive population of neoblasts, it remains unclear how flatworms dedicate a fraction of these neoblasts to adult neurogenesis. The vast majority of these cells express *piwi-1*, a member of the PIWI/Argonaute family of proteins (Reddien et al., 2005; van Wolfswinkel, 2014), and are constantly dividing (Newmark and Sánchez Alvarado, 2000). Recent studies have begun to tease apart the heterogeneity that exists within this once seemingly homogenous population of adult stem cells. Wagner and colleagues demonstrated, through single-cell transplants into lethally irradiated hosts, that there exists a sub-population of pluripotent neoblasts (cNeoblasts) capable of generating every tissue in the adult worm (Wagner et al., 2011). The discovery of the cNeoblast suggested that a cellular hierarchy existed within the neoblasts and that stem cell heterogeneity was likely underlying the diverse array of cellular lineages present in the flatworm. Further exploration of neoblast heterogeneity led to the classification of the (zeta)ζNeoblasts, which appear to be restricted to generate epidermal lineages. Additional analysis demonstrated the presence of non-ζNeoblasts, known as (sigma)σNeoblasts, which may contain the cNeoblast population due to the ability of the σNeoblasts to regenerate the ζNeoblast population (van Wolfswinkel et al., 2014). Lastly, (gamma)γNeoblasts were identified as a molecularly-distinct subpopulation of neoblasts, which express gut markers (van Wolfswinkel et al., 2014).

Despite the heterogeneity that has been observed in the neoblast population, the presence of a *bona fide* neural stem cell has not yet been demonstrated. A study employing a single-cell genomics approach investigating neoblast heterogeneity in the head of the animal identified the (nu)νNeoblasts, a class of neoblast that exhibits neural gene expression (Molinaro and Pearson, 2016). Through the use of an *in silico* lineage tracing technique, known as waterfall (Shin et al., 2015), gene markers enriched in the νNeoblasts were identified and detected in a subpopulation of dividing stem cells adjacent to the brain (Molinaro and Pearson, 2016). Although, functional studies remain to be done to cement the νNeoblast as a true neural stem cell, the presence of a dividing neoblast population with neural character that can be found directly adjacent to the brain, a putative neurogenic zone, is suggestive that a *bona fide* neural stem cell does exist in the planarian. It will be interesting to see whether there are multiple populations of neuronal stem cells, or if the νNeoblast is responsible for generating the diversity of neural subtypes found in the planarian brain. Regardless, νNeoblasts may represent a mechanism by which planarians dedicate a sub-fraction of their neoblasts toward adult neurogenesis.

## Neurogenic zones: how is adult neurogenesis dynamically modulated?

Upon amputation the planarian is able to rapidly re-establish its head and generate a fully functional brain without any pieces of the original tissue. To remake its brain the planarian must (1) form a blastema, (2) define a neurogenic zone and generate the brain primordium, (3) properly pattern newly generated brain tissue, (4) form the proper neural connections, and (5) functionally integrate the new tissue into existing neural tissue (Agata and Umesono, 2008). Exactly what causes the brain primordium to form is currently unknown, but several conserved developmental signaling pathways act to create a signaling landscape in the head that is permissive for neurogenesis. Briefly, the planarian must first re-establish its anterior-posterior (AP) axis which is accomplished via canonical WNT (Gurley et al., 2008) and Hedgehog (Hh) (Rink et al., 2009) signaling. After the anterior pole is established, anterior WNT inhibition (Petersen and Reddien, 2011), FGF signaling (Cebrià et al., 2002) and BMP signaling (Molina et al., 2007) create an environment that is permissive for the formation of the brain primordium in the ventral anterior of the head (Figure 1). This permissive signaling landscape must be maintained in the intact flatworm because disruption of any of these signaling pathways can lead to diminished neurogenesis, such as in *apc*(RNAi), or ectopic neurogenesis, as is observed in β–*catenin*(RNAi), *ndk*(RNAi), and *bmp*(RNAi) (Cebrià et al., 2002; Molina et al., 2007; Gurley et al., 2008). It is unclear exactly how ectopic neurogenesis is triggered, but it is clear that when the planarian neoblasts are not properly controlled, neural tissue can form in improper locations. The link between the signaling landscape and brain neurogenesis suggests either (1) that the neoblast population is poised for neurogenesis in all regions of the animal but must be prevented from acquiring a neural fate, or (2) that a subset of neoblasts can be biased toward neural fates through positional signaling. Further defining neurogenic zones and characterizing how they form *de novo* will be vital to our understanding of how the flatworm integrates its unlimited capacity for adult neurogenesis with the ability to dynamically scale, pattern and regenerate a complex neural tissue from a pool of adult stem cells.

The adult planarian brain is a highly plastic and dynamic structure. Not only is the entire brain turned over with new neurons arising from the neoblast compartment continuously, but the brain is constantly being scaled and patterned during periods of growth and degrowth (Baguñà, 2012). This raises the question of how planarians are able to dynamically coordinate the number of new neurons that are born vs. those that must be turned over and removed. Several WNT molecules are involved in scaling the brain relative to body size. *wnt5* has been shown to pattern the mediolateral axis through repression of medial fates and acts to restrict lateral brain growth while *wnt11-6* acts to restrict brain length along the AP axis (Gurley et al., 2010; Hill and Petersen, 2015). *wnt11-6*, which is expressed in posterior brain neurons, forms a spatial feedback loop through the induction of its inhibitor, *notum*, in anterior brain commissural neurons allowing the animal to dynamically regulate the length of its brain along the AP axis (Hill and Petersen, 2015). The feedback loop formed between *wnt11-6* and *notum* affects brain size through a neoblast-dependent mechanism and not through cell death (Hill and Petersen, 2015). *wnt11-6(RNAi)* animals possess a posteriorly-expanded brain and a concomitant increase in the number of brain progenitor cells during tissue remodeling while *notum(RNAi)* animals exhibit the opposite effect (Hill and Petersen, 2015), indicating that this spatial feedback loop somehow influences the generation of new neurons from the neoblast population to control brain length.

**Figure 2:**
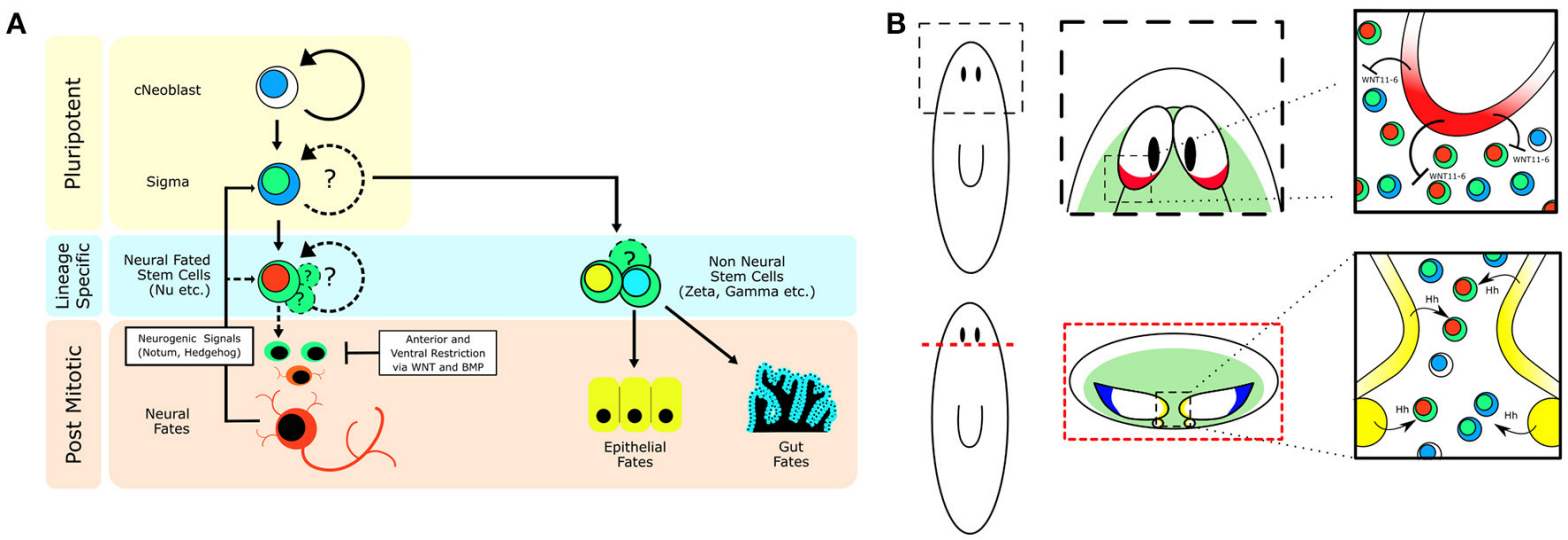
**Stem cell heterogeneity and conserved developmental signaling define planarian neurogenic zones. (A)** Cartoon showing how a population of pluripotent adult stem cells (cNeoblasts) are capable of generating stem cell heterogeneity and a multitude of differentiated cell types in the adult flatworm. Emphasis on how environmental signaling restricts cellular fate. **(B)** Depiction of planarian neurogenic zones near the brain, which involve signaling between mature brain neurons and nearby stem cells to coordinate adult neurogenesis and brain patterning. (Top—Top down view of the planarian brain, anterior is up; Bottom—Cross section of the planarian brain, dorsal is up.) Green zones illustrate where adult stem cells can be found. The red brain region is a source of WNT11-6, which restricts brain length through signaling to the stem cells. The yellow region denotes the ventral-medial brain and nerve cords which express Hh and signal to nearby stem cells to promote adult neurogenesis. The blue region defines a unique dorsal-lateral zone of the brain that possesses several cell types, of which the progenitors are currently unknown. Black ovals denote the planarian anterior photoreceptors (eyes).

If neurons are expressing signaling molecules to dynamically shape the brain through communication with stem cells, then stem cells must express the signal-transduction machinery in order to respond. A recent study on Hedgehog (Hh) signaling demonstrated its role in directing adult neurogenesis through signaling from neurons to the stem cell compartment (Currie et al., 2016). The authors found that the Hh ligand was primarily expressed by cholinergic neurons in the ventral-medial (VM) region of the brain. Interestingly, decreased Hh signaling results in animals that exhibit fewer neural progenitor cells and fewer newly born VM acetylcholine neurons, whereas increased Hh signaling increases the number of several types of neural progenitors (Currie et al., 2016). Currie and colleagues then further employed single-cell RNA-sequencing data (Molinaro and Pearson, 2016) specifically from head stem cells to demonstrate that many of these stem cells expressed both WNT and Hh signal transduction machinery suggesting they could be receptive to signaling cues from the brain (Currie et al., 2016). Both WNT and Hh signaling appear capable of influencing neoblasts close to the brain. It is currently unclear which neoblasts are being influenced, but with Hh expression being confined to the VM nervous system it would stand to reason that neoblasts which receive this message are in the pocket of cells between the brain lobes and nerve cords (Figure 2). It remains unknown whether stem cells that contribute to the brain do so in a regionally biased manner, or if any neoblast flanking the brain is free to contribute new neurons when signaled to do so. Hedgehog signaling affects VM neuronal fates, but what signaling directs dorsal-lateral (DL) fates is unclear. There is a large range of neuronal subtypes (*cintillo*+, *gpas*+, *chat*+, *gad*+, and many others; Oviedo et al., 2003; Nishimura et al., 2008, 2010; Iglesias et al., 2011) located in the DL regions of the brain and determining what signals direct DL neoblasts (Figure 1C) to integrate into this region and acquire these fates will be important in understanding the full link between mature brain neurons and nearby stem cells. From the single-stem cell RNAseq data, the complex “code” of over 10 WNT receptors (Gurley et al., 2008), the frizzled genes, are very heterogeneously expressed, suggesting more heterogeneous responses to WNT signals than with the Hh pathway (Currie et al., 2016). In the future, it will be interesting to tease apart how local WNT signals direct stem cells toward specific cell fates, perhaps through the action of various TCF/LEF transcription factors.

## Conclusions

The planarian is unique in its ability to maintain and regenerate its full adult brain continuously and is a powerful model with which to study adult neurogenesis in an *in vivo* context. In order to maintain continuous and unlimited adult neurogenesis the planarian employs a robust and self-organizing landscape of developmental signaling pathways to link mature neuronal tissue with nearby populations of adult stem cells, ensuring the controlled generation and integration of new neurons that can scale in real time. Coupled with the planarian's ability to instruct the generation of a brain primordium *de novo* after complete tissue loss, the flatworm is able to both regenerate and maintain its entire brain without limits. Underlying this dynamic link between mature neurons and stem cells, the neoblasts are capable of generating every neuronal subtype found in the adult flatworm. Additional studies into the full heterogeneity of the adult stem cells and the lineages between stem cell and neuron are still needed to fully understand how planarians are able to direct and control unlimited adult neurogenesis without negative consequences to the organism. Although, much remains to be discovered, the planarian turns the idea of a fixed brain on its head and raises the larger question of whether this total brain plasticity is possible in other organisms such as humans.

## Author contributions

BP and DB wrote and edited the manuscript.

## Funding

DB was funded by Natural Sciences and Engineering Research Council award # RGPIN-2016-06354. BP was funded by Ontario Institute for Cancer Research award #IA-026.

### Conflict of interest statement

The authors declare that the research was conducted in the absence of any commercial or financial relationships that could be construed as a potential conflict of interest. The reviewer AB and handling Editor declared their shared affiliation, and the handling Editor states that the process nevertheless met the standards of a fair and objective review.
